# Betulin Alleviates 5-Fluorouracil-Induced Intestinal Mucositis in Mice

**DOI:** 10.3390/nu18152487

**Published:** 2026-08-01

**Authors:** Shiyi Sun, Ziming Wang, Haoyue Xu, Chengheng Dai, Wenhannian Li, Mengcheng Ying, Ayimnisa Ayitniyaz, Changmin Shao, Zhiwei Hu

**Affiliations:** 1 National and Local Joint Engineering Research Center of Ecological Treatment Technology for Urban Water Pollution, Zhejiang Provincial Key Laboratory for Water Environment and Marine Biological Resources Protection, College of Life and Environmental Science, Wenzhou University, Wenzhou 325035, China; 15158344735@163.com (S.S.); 20200086@wzu.edu.cn (Z.W.); 18705826602@163.com (H.X.); 13656656387@163.com (C.D.); 13185968070@163.com (W.L.); 18157565916@163.com (M.Y.); 15001432683@163.com (A.A.); 2 Wenzhou Institute, University of Chinese Academy of Sciences, Wenzhou 325001, China

**Keywords:** betulin, 5-fluorouracil, chemotherapy-induced intestinal mucositis, inflammation, gut microbiota

## Abstract

**Background:** Chemotherapy-induced intestinal mucositis (CIM) is a common gastrointestinal complication of anticancer therapy, and 5-fluorouracil (5-FU) is among the agents most frequently implicated. Betulin (BE), a lupane-type pentacyclic triterpenoid with anti-inflammatory and cytoprotective activities, has not been systematically evaluated in 5-FU-induced CIM. **Purpose:** This study evaluated the therapeutic effects of BE in a mouse model of 5-FU-induced CIM and examined associated histopathological, apoptotic, inflammatory, and gut microbiota changes. **Methods:** BALB/c mice received intraperitoneal 5-FU (30 mg/kg/day) for four consecutive days, followed by oral BE at 0, 50, 100, 200, or 400 mg/kg/day for another four days. Clinical manifestations, colon morphometry, blinded descriptive histopathological assessment, apoptosis- and inflammation-related markers, and gut microbiota were assessed. **Results:** BE treatment alleviated body weight loss, diarrhea, and reduced food intake and was associated with improved colon morphometry and less severe histological injury. At 200 mg/kg, BE treatment was associated with lower *Tnf* and *Nos2* mRNA expression, lower Bax and total caspase-3 protein abundance, higher Bcl-2 protein abundance, and lower iNOS protein abundance. Microbiota analysis of the 200 mg/kg group showed treatment-associated differences in microbial diversity and community composition; these findings were considered exploratory because of the small sample size. **Conclusions:** BE showed therapeutic potential in 5-FU-induced intestinal mucositis. The molecular and microbiota findings obtained at 200 mg/kg support associations with apoptosis-, inflammation-, and microbiota-related changes but do not establish a definitive mechanism.

## 1. Introduction

Chemotherapy remains indispensable in the treatment of many malignancies, but gastrointestinal toxicity continues to limit its tolerability [1,2,3]. Among these toxicities, chemotherapy-induced intestinal mucositis (CIM) is especially common. Approximately 40% of patients receiving standard chemotherapy develop this complication, which is characterized by diarrhea, mucosal inflammation, crypt injury, and barrier dysfunction [4]. Patients may also experience abdominal discomfort, malabsorption, weight loss, and reduced physical strength [5,6]. These changes often compromise quality of life and can lead to treatment interruption, dose reduction, or discontinuation. Although 5-FU is widely used because it blocks DNA synthesis in tumor cells, it also damages rapidly renewing intestinal epithelium, thereby promoting epithelial apoptosis [7] and excessive inflammatory signaling [8]. Together, these events contribute directly to mucosal breakdown. The gut microbiota has also emerged as an important factor in CIM because of its role in epithelial barrier maintenance and immune homeostasis [9,10]. In patients with mucositis, loss of microbial diversity and depletion of beneficial taxa are common [11,12], and these alterations may aggravate systemic inflammation and disease severity.

At present, management of 5-FU-induced CIM is still mainly supportive. Commonly used measures, including somatostatin analogs for diarrhea and glutamine-containing preparations for mucosal repair, provide only partial relief and do not adequately address the biological basis of the disease [13]. This unmet need has prompted interest in natural compounds with mucosal protective activity. Terpenoids are particularly attractive because many members of this class show anti-inflammatory, hepatoprotective, antitumor, and microbiota-modulating properties [14,15]. Betulin (BE), a natural pentacyclic triterpenoid isolated from birch bark [16], has long been used for inflammatory skin lesions and burns and is described in Traditional Chinese Medicine as clearing heat, dispelling dampness, reducing swelling, and relieving pain [17]. Modern pharmacological studies have further demonstrated anti-inflammatory, hepatoprotective, antitumor, and immunomodulatory effects of BE [18,19,20,21]. Compared with some derivatives, BE is readily obtainable in large amounts from birch bark and has shown favorable gastrointestinal protective activity in experimental settings [22,23,24]. Nevertheless, whether BE can protect against CIM, and by what mechanisms, has not been systematically examined.

**Figure 1:**
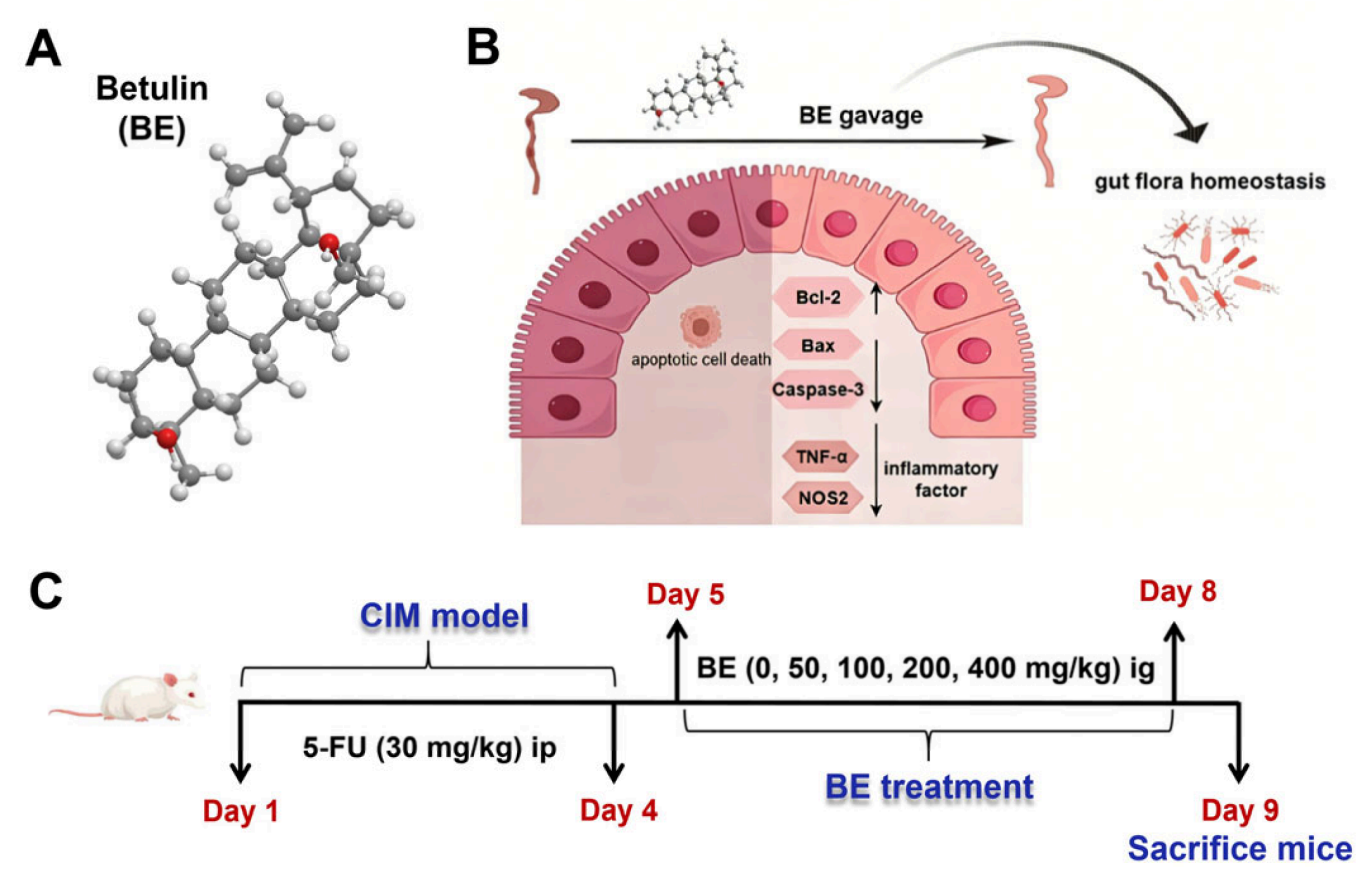
(**A**) Chemical structure of BE; (**B**) Schematic summary of the inflammatory, apoptotic, and microbiota-related changes associated with BE treatment in 5-FU-induced intestinal mucositis; (**C**) Schematic overview of the animal experiment and treatment schedule (ip, intraperitoneal; ig, intragastric).

Accordingly, we established a 5-FU-induced mouse model of intestinal mucositis to evaluate the short-term overt toxicity and therapeutic potential of BE (Figure 1). After assessing clinical and histopathological outcomes across multiple doses, the 200 mg/kg dose was selected as a representative treatment for exploratory molecular and microbiota analyses. At this dose, apoptosis- and inflammation-related markers were assessed in colonic tissue, and gut microbial diversity and composition were profiled to determine whether these changes were associated with the observed therapeutic response.

## 2. Materials and Methods

### 2.1. Reagents

BE (purity ≥ 98%), 5-FU, cottonseed oil, and paraformaldehyde were obtained from Shanghai Source Leaf Biotechnology Co., Ltd. (Shanghai, China). RIPA lysis buffer was obtained from Beijing Biotopped Biotechnology Co., Ltd. (Shanghai, China). The BCA Protein Assay Kit was obtained from Jiangsu CWBIO Biotechnology Co., Ltd. (Taizhou, China). H&E staining reagents were purchased from Fuzhou Weibokang Biotechnology Co., Ltd. (Fuzhou, China). The PAGE Gel Rapid Preparation Kit was obtained from Nanjing Novizan Biotechnology Co., Ltd. (Nanjing, China). TEMED, protein markers, PBS, and TBST were purchased from Beijing Thermo Fisher Biochemical Products Co., Ltd. (Beijing, China). ECL reagent was obtained from Dalian Meilun Biotechnology Co., Ltd. (Dalian, China). Primary antibodies against β-actin, Bax, Bcl-2, total caspase-3, and iNOS were obtained from ABclonal Biotechnology Co., Ltd. (Wuhan, China). Primers for *Tnf*, *Nos2*, and *Gapdh* were synthesized by Sangon Biotech (Shanghai, China) Co., Ltd. RNA isolation and reverse-transcription reagents were purchased from Yeasen Biotechnology (Shanghai, China) Co., Ltd.

### 2.2. Animals

Specific-pathogen-free male BALB/c mice aged 5–8 weeks were supplied by Beijing Vital River Laboratory Animal Technology Co., Ltd. (Beijing, China). Animals were maintained at 22 ± 2 °C and 50 ± 5% relative humidity under a 12 h light/12 h dark photoperiod, with free access to chow and water. All procedures were approved by the Animal Ethics Committee of Wenzhou University and were carried out in accordance with institutional and national guidelines for laboratory animal care (approval no. WZU-2026-034).

### 2.3. Safety Assessment of BE

For the safety evaluation, mice were randomly assigned to six experimental groups via computer-generated randomization (*n* = 4/group): blank, control, and BE groups receiving 50, 100, 200, or 400 mg/kg/day. BE was suspended in cottonseed oil and administered by oral gavage once daily for nine consecutive days. Animals were observed closely during the first 2 h after each administration and monitored daily thereafter. Food intake, water intake, body weight, and general condition were recorded throughout the study. At the endpoint, the animals were euthanized, and colonic samples were collected for gross and histological assessment of short-term overt toxicity.

### 2.4. Induction of CIM and Treatment Protocol

After a one-week acclimation period, mice were randomized using a computer-generated allocation sequence into six groups (*n* = 4 per group): control, 5-FU model, and four BE treatment groups receiving 50, 100, 200, or 400 mg/kg/day. All groups except the control group received intraperitoneal 5-FU (30 mg/kg/day) on Days 1–4 to induce intestinal mucositis; control mice received an equivalent volume of normal saline. From Days 5–8, BE was administered once daily by oral gavage at the indicated doses. Control and model mice received cottonseed oil on the same schedule. Body weight, stool appearance, food intake, water intake, and general condition were recorded daily.

### 2.5. Disease Activity Index (DAI) Scoring

DAI scores were assessed once daily by two investigators blinded to group allocation, which remained concealed until data collection was complete, according to the method described by Qiu et al. [25]. The DAI comprised stool consistency and body weight loss scores as follows:

- Stool consistency: 0, normal well-formed pellets; 1, mildly moist stool; 2, loose, unformed stool with moderate perianal staining; 3, watery stool with severe perianal contamination; 4, death.
- Body weight loss: 0, no loss; 1, 1–5%; 2, 6–10%; 3, 11–15%; 4, >15%.

The daily DAI score for each mouse was calculated as the mean of the stool consistency and body weight loss scores.

### 2.6. Histopathological Analysis

After euthanasia, colon length and width were recorded. Colonic tissues were fixed in 4% paraformaldehyde for 24 h, dehydrated through a graded ethanol series, cleared in xylene, embedded in paraffin, and sectioned at 5 μm. Sections were stained with H&E and examined by light microscopy. Crypt depth was measured using ImageJ (1.54g) in well-oriented crypts from each animal. Measurements from the evaluable crypts were averaged to obtain one animal-level value for statistical analysis.

A representative H&E-stained colonic section was evaluated by an independent pathologist blinded to group identity using a predefined, modified semi-quantitative framework for colonic injury [26]. The assessment included crypt architectural distortion, crypt regeneration, crypt loss, luminal sloughing of cellular debris, inflammatory-cell infiltration in the lamina propria, and mucosal ulceration. Each parameter was assigned a descriptive severity score, and the parameter scores were summed to obtain an aggregate score for the representative section.

### 2.7. Western Blot Analysis

Colon tissues were homogenized in RIPA buffer containing protease inhibitors to obtain total protein. Protein concentration was measured with the BCA assay. Equal protein aliquots (20 μg) were separated on 10% SDS-PAGE gels and transferred to PVDF membranes. Membranes were blocked in 5% skim milk prepared in TBST for 2 h at room temperature and then incubated overnight at 4 °C with primary antibodies against β-actin (1:1000), Bax (1:20,000), Bcl-2 (1:2000), iNOS (1:2000) and total caspase-3 (1:2000). After TBST washes, membranes were incubated with appropriate secondary antibodies (1:2000) for 1 h at room temperature. Immunoreactive bands were detected with ECL reagent and quantified in ImageJ. Protein abundance was expressed relative to β-actin.

### 2.8. Quantitative Real-Time PCR (qRT-PCR) Analysis of Inflammatory Cytokines

Total RNA was isolated from colonic tissue using the Flash Cell/Tissue Total RNA Kit purchased from Yeasen Biotechnology (Shanghai, China) Co., Ltd. according to the manufacturer’s instructions. RNA concentration and purity were assessed by spectrophotometry. Reverse transcription was performed using the Hifair^®^ AdvanceFast 1st Strand cDNA Synthesis Kit (No Dye) purchased from Yeasen Biotechnology (Shanghai, China) Co., Ltd. at 50 °C for 15 min and 85 °C for 5 s. The resulting cDNA was diluted 1:5 with nuclease-free water and stored at −20 °C until use.

Each 20 μL qPCR reaction contained 10 μL of 2× SYBR Green Master Mix, 0.4 μL of forward primer (10 μM), 0.4 μL of reverse primer (10 μM), 2 μL of diluted cDNA, and 7.2 μL of nuclease-free water. A no-template control was included in each run. The amplification protocol consisted of 95 °C for 30 s, followed by 40 cycles of 95 °C for 5 s and 60 °C for 30 s. Melt-curve analysis was performed from 65 °C to 95 °C in 0.5 °C increments to confirm amplification specificity. Relative gene expression was calculated using the 2^−ΔΔCt^ method after normalization to *Gapdh*. The primer sequences used for qRT-PCR are provided in Table S3.

### 2.9. Gut Microbiota Analysis

On Day 9, fecal samples were collected from three mice per group (five fecal pellets per mouse). Total genomic DNA was isolated using the MagPure Soil DNA LQ Kit (Magen, Guangzhou, China) according to the manufacturer’s protocol. DNA concentration and integrity were assessed using a NanoDrop 2000 spectrophotometer (Thermo Fisher Scientific, Waltham, MA, USA) and agarose gel electrophoresis. Extracted DNA was stored at −20 °C until further analysis. The extracted DNA served as the template for PCR amplification of bacterial 16S rRNA gene fragments with barcode-indexed primers and Takara Ex Taq DNA polymerase (Takara). For bacterial diversity analysis, the V3-V4 variable region was amplified with primers 343F (5′-TACGGRAGGCAGCAG-3′) and 798R (5′-AGGGTATCTAATCCT-3′).

Amplicon quality was verified by agarose gel electrophoresis. PCR products were purified using AMPure XP magnetic beads (Agencourt, Indianapolis, IN, USA) and subjected to a second round of PCR. After an additional purification step with AMPure XP beads, the final amplicons were quantified using the Qubit dsDNA Assay Kit (Thermo Fisher Scientific, USA). Normalized amplicon libraries were sequenced on an Illumina NovaSeq 6000 platform (Illumina Inc., San Diego, CA, USA) to generate 250 bp paired-end reads (OE Biotech Co., Ltd., Shanghai, China).

Library sequencing and initial data processing were performed by OE Biotech Co., Ltd. (Shanghai, China). Raw FASTQ reads were processed using Cutadapt to remove adapter sequences. After quality trimming, paired-end reads were denoised, merged, and screened for chimeras using DADA2 within QIIME 2 [27]. Microbial community analyses were performed in QIIME 2 and R. Alpha diversity was assessed using the Shannon and Simpson indices. Beta diversity was visualized by PCoA, PCA, and NMDS based on Binary Jaccard and weighted UniFrac distances, and between-group differences were assessed by PERMANOVA. LEfSe was used for exploratory identification of candidate differentially abundant taxa. Given the limited sample size, all microbiota analyses were interpreted as exploratory and hypothesis-generating.

### 2.10. Statistical Analysis

Data are expressed as mean ± SD and were analyzed using SPSS 22.0. Depending on data distribution, group comparisons were carried out using one-way ANOVA followed by Tukey’s post hoc test. A value of *p* < 0.05 was regarded as significant.

## 3. Results

### 3.1. Safety Profile of BE in Mice

**Figure 2:**
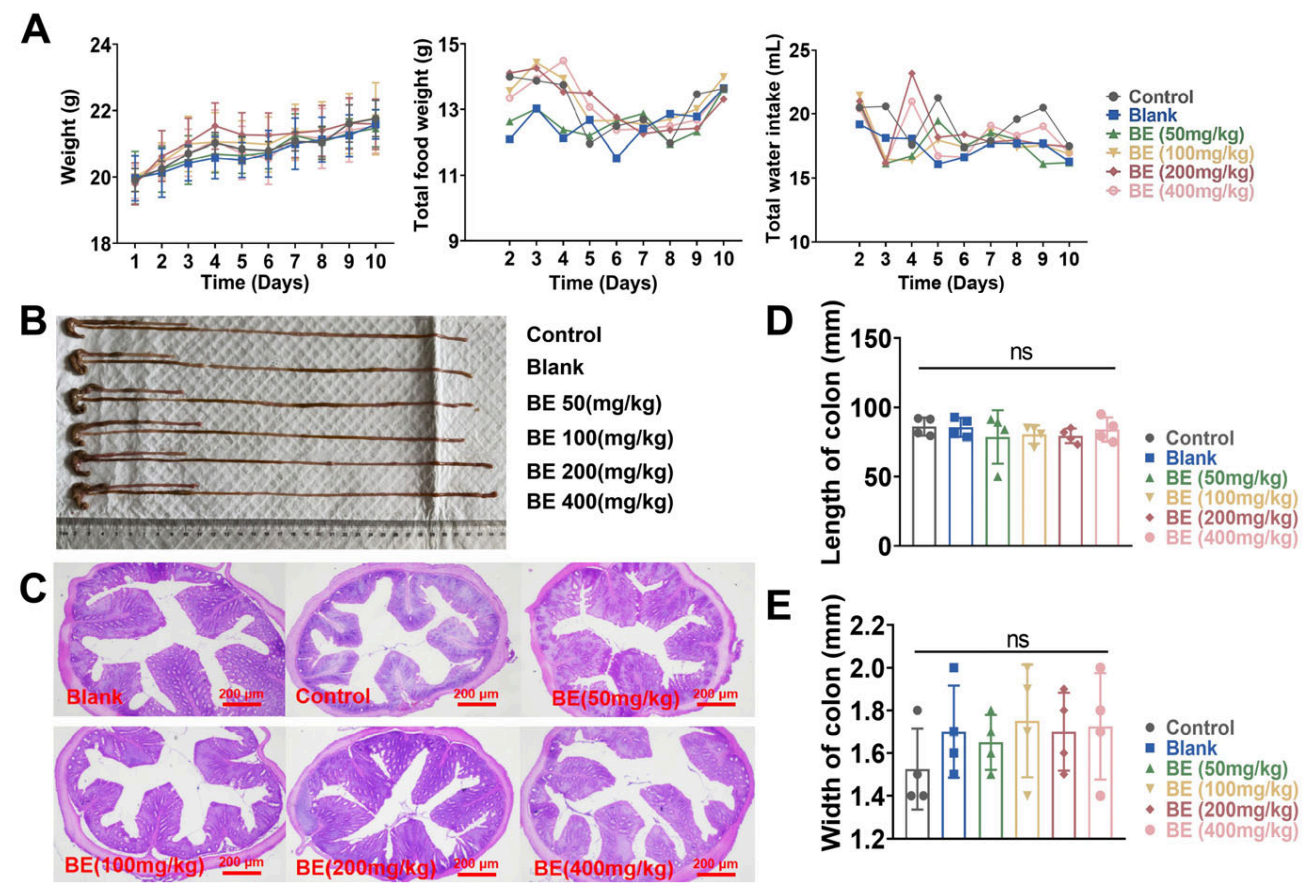
Safety assessment of BE in mice. (**A**) Body weight, cumulative food intake, and cumulative water intake; (**B**) Representative gross images of intestinal tissue; (**C**) Representative H&E-stained colon sections (×10); (**D**) Colon length; and (**E**) Colon width. (*n* = 4). “ns” means not significant.

To assess short-term overt toxicity, body weight, food and water intake, general condition, and intestinal morphology were monitored throughout the observation period. No deaths occurred in any BE-treated group, including the 400 mg/kg group. No overt changes in coat appearance, respiration, fecal output, posture, or spontaneous feeding were observed. Body weight, food intake, and water intake did not differ significantly among groups (Figure 2A; *p* > 0.05).

Gross examination revealed no treatment-related abnormalities in the colon (Figure 2B). Colon length and width were comparable among groups (Figure 2D,E; *p* > 0.05). H&E-stained sections showed preserved crypt organization, an orderly epithelial layer, and no clear inflammatory-cell infiltration in BE-treated mice (Figure 2C). Blinded semi-quantitative scoring showed no increase in mucosal injury relative to the control group (Table S1). In addition, no obvious gross anatomical abnormalities were observed in the heart, liver, spleen, lungs, or kidneys (Figure S1). Taken together, no overt adverse effects were detected after oral BE administration at doses up to 400 mg/kg under the conditions and duration of this study.

### 3.2. BE Ameliorates 5-FU-Induced CIM Symptoms

Body weight, food intake, water intake, and stool consistency were recorded daily as indicators of overall condition during model induction and treatment. Following 5-FU exposure, mice developed weight loss and diarrhea, findings consistent with intestinal injury and impaired feeding. These measures were used to evaluate the therapeutic response to BE.

**Figure 3:**
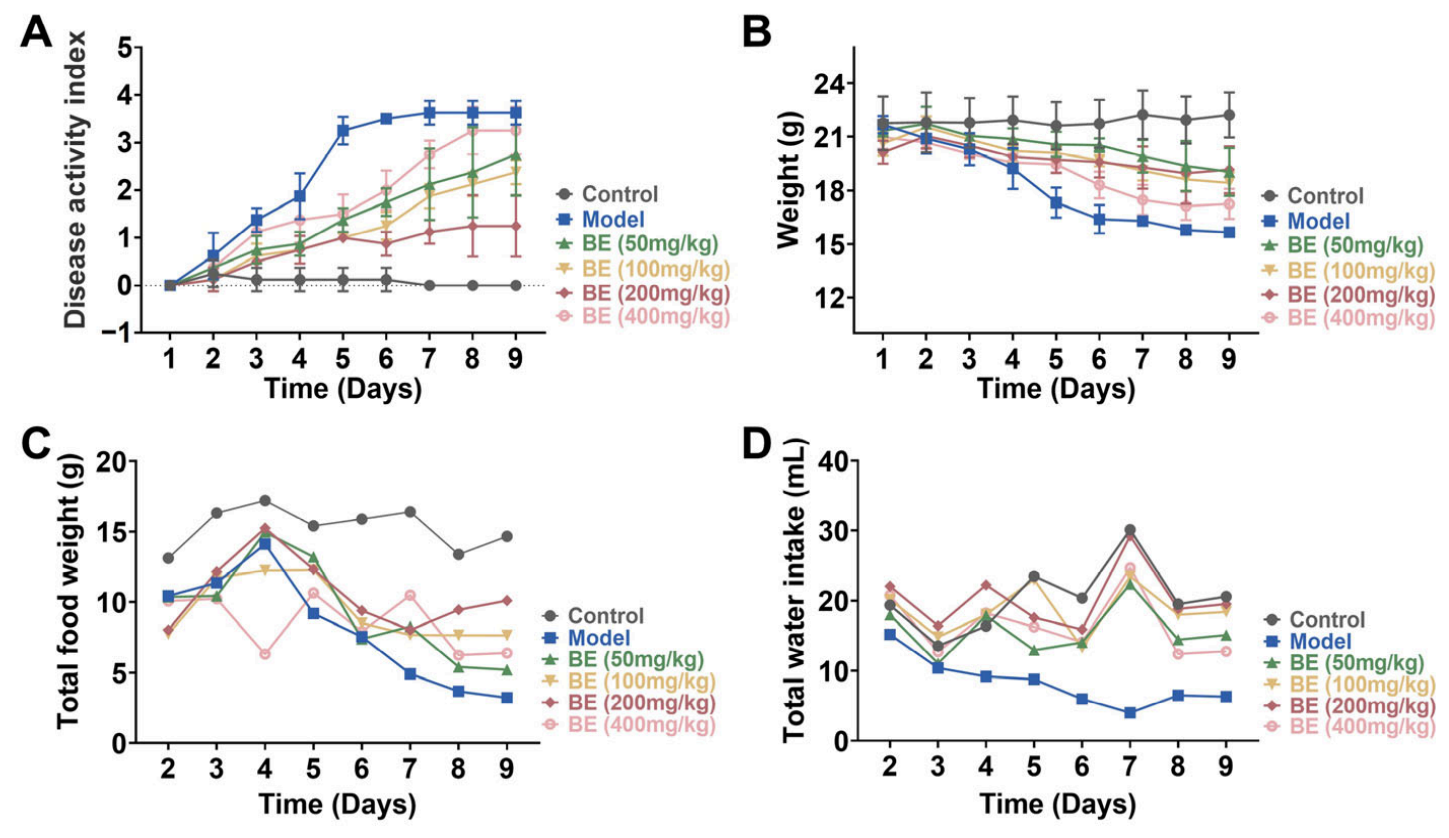
Effects of BE on clinical manifestations of 5-FU-induced mucositis. (**A**) Disease activity index; (**B**) Body weight; (**C**) Total food intake; (**D**) Total water intake. (*n* = 4).

As shown in Figure 3, control mice maintained smooth fur, normal feeding and drinking, and formed stools. In contrast, mice receiving 5-FU (30 mg/kg, i.p.) developed diarrhea, reduced food intake, decreased activity, rough fur, and, in some cases, a hunched posture from Day 2 onward, with greater severity between Days 3 and 5. BE treatment ameliorated these manifestations to varying degrees, with more evident improvement in the 100, 200, and 400 mg/kg groups.

Consistent with these observations, 5-FU-treated mice lost body weight from Days 3–7, whereas control mice continued to gain weight (Figure 3B). BE treatment was associated with less body weight loss, lower DAI scores, and partial improvement in food and water intake (Figure 3).

**Figure 4:**
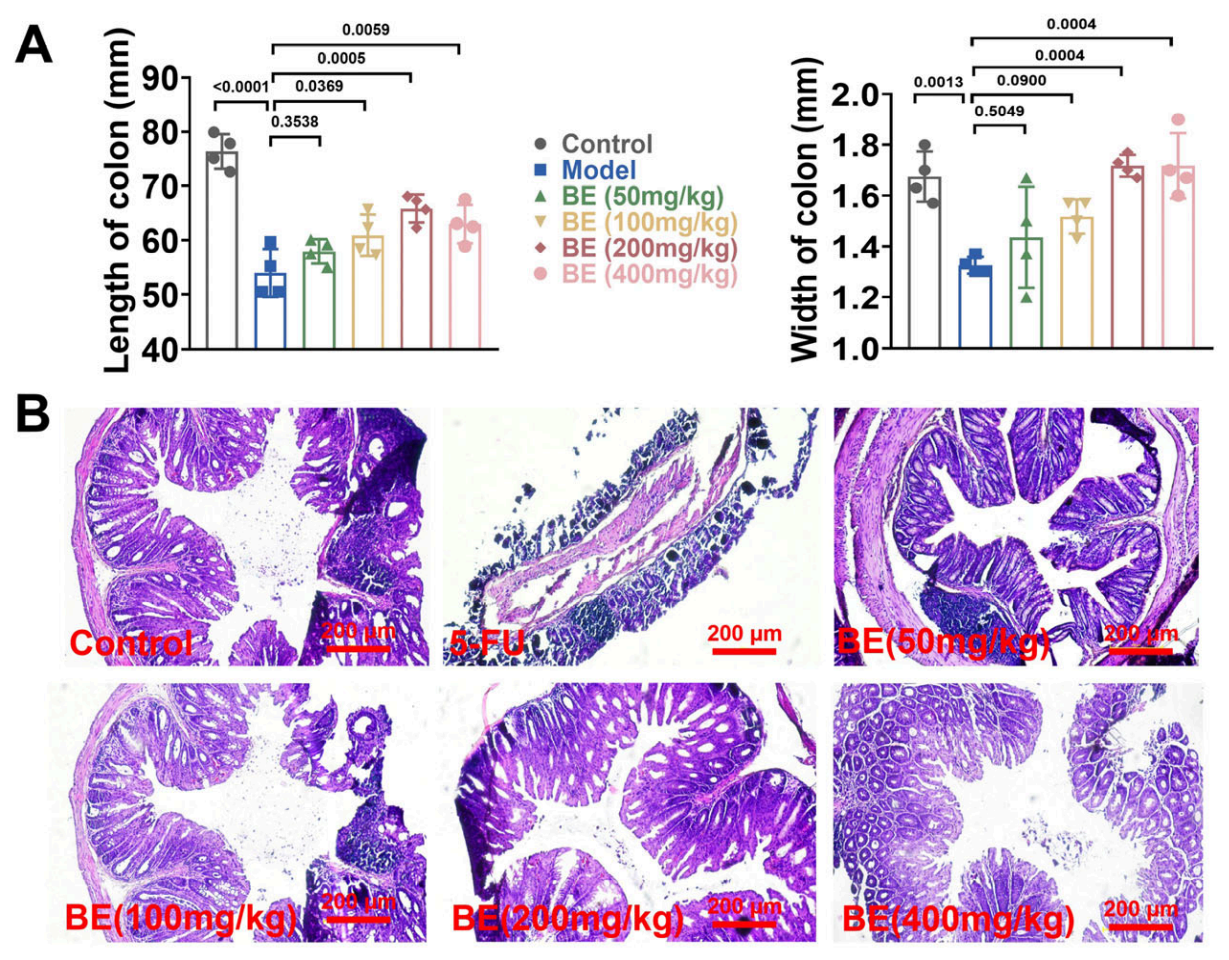
Morphometric and histopathological effects of BE in 5-FU-treated mice. (**A**) Colon length and width; (**B**) Representative H&E-stained colon sections (×10). (*n* = 4).

Morphometric and histopathological analyses provided convergent evidence that BE attenuated 5-FU-induced colonic injury. As shown in Figure 4A, 5-FU reduced colon length and width, whereas BE treatment partially reversed these changes. Quantifiable, well-oriented crypts were observed in the control and BE-treated groups, and crypt-depth measurements are presented in Figure S2. In the 5-FU model group, severe mucosal destruction and extensive crypt loss prevented reliable crypt-depth measurement. Therefore, the crypt-depth data described the differences between the control group and the BE-treated groups. Consistent with these morphometric findings, H&E-stained colon sections from the 5-FU group showed disruption of crypt architecture, inflammatory-cell infiltration in the lamina propria, vacuolization, and edema involving the mucosal and muscular layers (Figure 4B). These abnormalities appeared less pronounced after BE treatment, with better-preserved crypt architecture and reduced inflammatory infiltration, edema, and vacuolization. Blinded semi-quantitative scoring further supported these observations: the aggregate injury score was higher in the model group than in the control group and was lower in the BE-treated groups (Table S2).

### 3.3. BE Treatment Is Associated with Changes in Apoptosis-Related Proteins in Colonic Tissue

**Figure 5:**
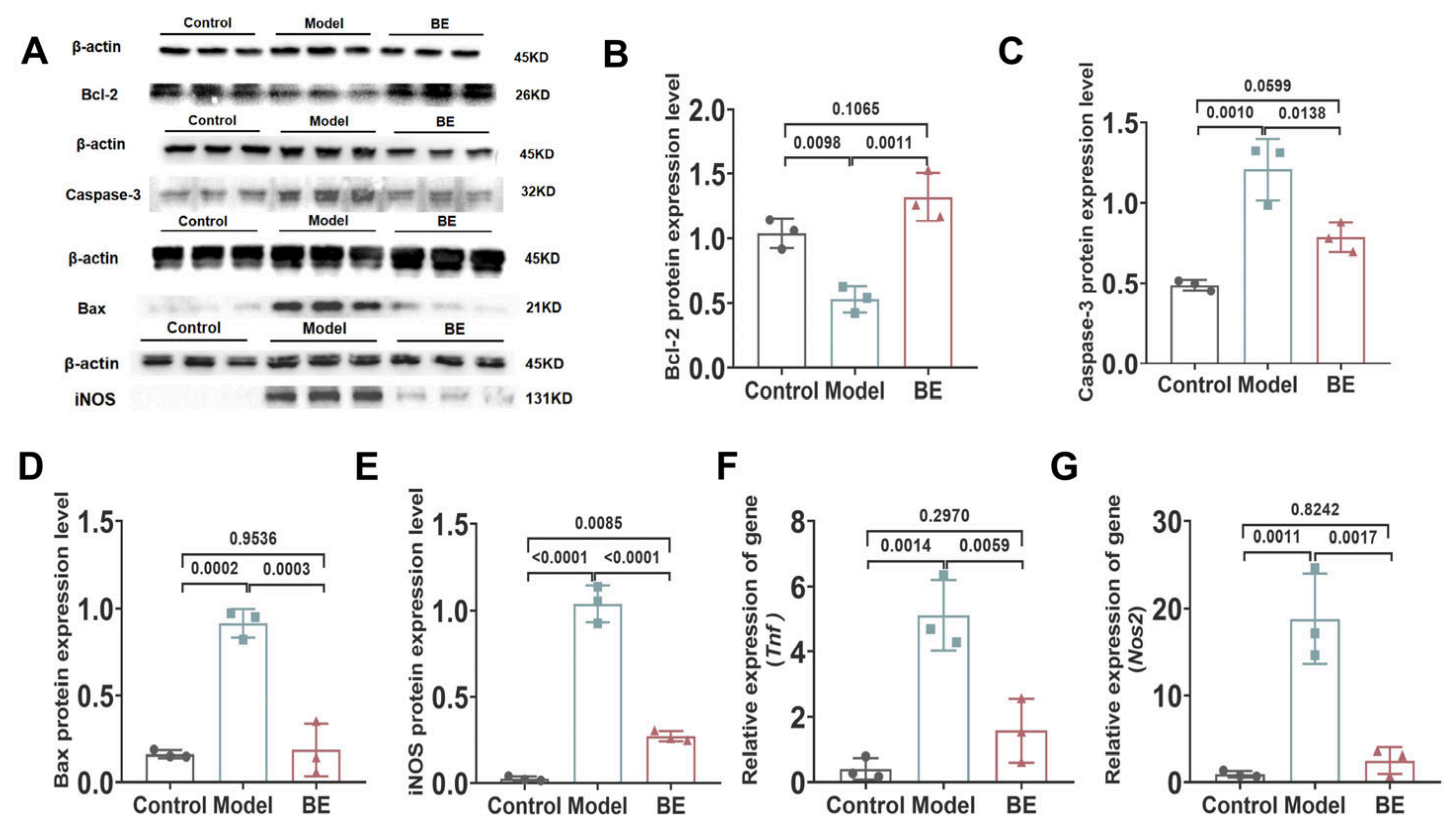
Effects of BE (200 mg/kg) on apoptosis- and inflammation-related markers. (**A**) Representative Western blot bands for Bcl-2, total caspase-3, Bax, iNOS, and β-actin. (**B**–**E**) Relative protein abundance of Bcl-2, total caspase-3, Bax, and iNOS. (**F**,**G**) Relative mRNA expression levels of *Tnf* and *Nos2*. Western blot and qRT-PCR analyses were performed using three independent biological samples per group. Three mice per group were selected from the available animals to ensure balanced group sizes across the molecular analyses.

Apoptosis-related proteins in colonic tissue were examined by Western blotting. As shown in Figure 5A–D, the 5-FU model group showed higher Bax and total caspase-3 abundance and lower Bcl-2 abundance than the control group. In the BE 200 mg/kg group, Bax and total caspase-3 abundance were lower, whereas Bcl-2 abundance was higher, than in the model group. These findings indicate that BE treatment at 200 mg/kg was associated with partial normalization of apoptosis-related protein expression. Triplicate technical experiments are supplemented in Figures S3–S7.

### 3.4. BE Attenuates 5-FU-Associated Inflammatory Marker Expression

At the transcript level, 5-FU treatment was associated with higher colonic *Tnf* and *Nos2* mRNA expression than that observed in the control group, whereas BE treatment at 200 mg/kg was associated with lower expression of both genes. Consistent with the *Nos2* transcript results, iNOS protein abundance was also lower in the BE-treated group than in the 5-FU model group (Figure 5E–G).

### 3.5. Gut Microbiota Changes Associated with BE Treatment

To characterize gut microbiota changes associated with BE treatment, fecal samples from the control, model, and BE 200 mg/kg groups were analyzed by 16S rRNA sequencing. The 200 mg/kg group was selected as the representative BE treatment group for microbiota analysis.

**Figure 6:**
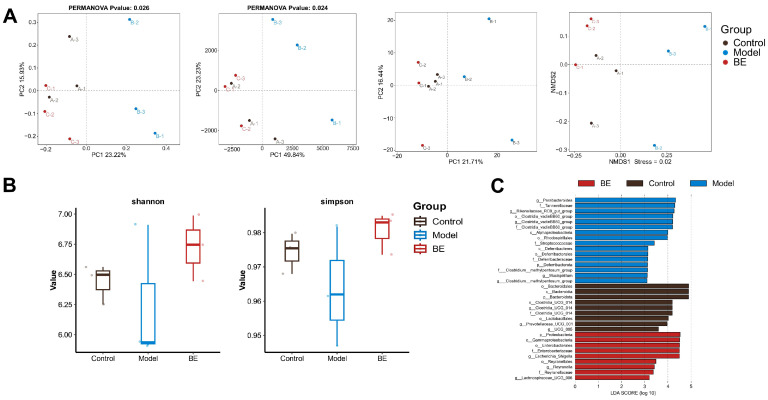
BE (200 mg/kg) modulates gut microbial diversity and composition in 5-FU-treated mice. (**A**) Beta-diversity plots generated by PCoA, PCA, and NMDS; (**B**) Alpha-diversity indices (Shannon and Simpson); (**C**) LEfSe results. (*n* = 3).

Ordination analyses based on PCoA, PCA, and NMDS (stress < 0.1) showed group-level clustering patterns, with the BE samples positioned closer to the control samples than to the model samples (Figure 6A). Given the small sample size, these patterns should be interpreted as exploratory.

Shannon and Simpson index values were lower in the model group than in the control and BE groups, whereas the BE group showed the highest values (Figure 6B). This pattern is consistent with higher alpha diversity after BE treatment but does not demonstrate complete normalization of the microbial community.

LEfSe analysis (LDA threshold > 2) suggested differentially abundant taxa among groups (Figure 6C). The control group was enriched in Bacteroidota, Bacteroidia, and Lactobacillales. In contrast, the model group showed enrichment of taxa associated with dysbiosis and inflammation, including Desulfobacterota and Parabacteroides. The BE group showed higher abundance of potentially beneficial taxa such as Lactobacillus, Lachnospiraceae, and Ruminococcaceae. Given the limited sample size, these taxonomic findings should be viewed as exploratory and hypothesis-generating.

**Figure 7:**
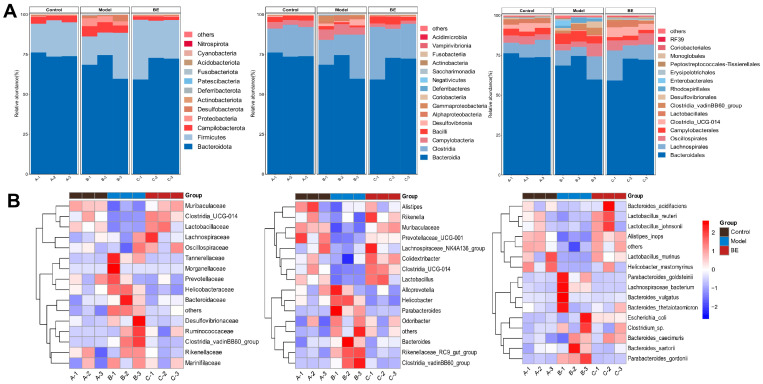
Taxonomic composition of the gut microbiota after BE (200 mg/kg) treatment. (**A**) Relative abundance at the phylum, class, and order levels; (**B**) Relative abundance at the family, genus, and species levels. (*n* = 3).

Analysis of community composition yielded a similar pattern. Samples from the control and BE groups clustered closely together and remained distinct from those of the model group (Figure 7A). At the phylum, class, and order levels, both the control and BE groups were dominated by Bacteroidota, Bacteroidia, and Bacteroidales, whereas the model group exhibited loss of Bacteroidota together with expansion of Firmicutes-related lineages, indicating marked re-structuring of the microbial ecosystem. Heatmap clustering yielded the same pattern, with BE and control samples grouping together and model samples forming an independent cluster (Figure 7B). These findings suggest that the remodeling of the gut microbiota was associated with the phenotypic improvements observed after BE treatment.

## 4. Discussion

CIM remains a clinically important toxicity that can interrupt treatment schedules and substantially impair patient well-being [1]. Although 5-FU is a cornerstone of many chemotherapy regimens, it readily injures the intestinal mucosa by promoting apoptosis in rapidly renewing epithelial cells and amplifying inflammatory signaling [6]. Because the intestinal epithelium depends on continuous renewal to maintain barrier integrity, it is particularly vulnerable to this insult. Current concepts therefore regard mucositis as a dynamic process involving epithelial injury, inflammatory amplification, attempted regeneration, and, when injury resolves, gradual structural recovery [28].

In the present study, BE was associated with improvement in a mouse model of 5-FU-induced intestinal mucositis. In the short-term overt toxicity assessment, no overt adverse effects were detected after oral BE administration at doses up to 400 mg/kg under the conditions used. Repeated 5-FU administration produced the expected clinical manifestations of mucositis, including diarrhea, reduced food and water intake, body weight loss, rough fur, and decreased activity [29]. These abnormalities were alleviated to varying degrees by BE treatment, supporting further evaluation of its therapeutic potential.

The morphometric and histopathological findings were consistent with the clinical observations. 5-FU reduced colon length and width and produced colonic mucosal injury characterized by crypt architectural disruption, inflammatory-cell infiltration, vacuolization, and edema. BE treatment was associated with lower total histopathological injury scores and partial preservation of crypt depth. The addition of blinded semi-quantitative scoring and animal-level morphometric analysis strengthens the tissue-level evidence. Nevertheless, the scoring system retains an observer-dependent component, and crypt-depth measurements depend on section orientation. The histological findings should therefore be interpreted together with the clinical and molecular readouts rather than as standalone proof of tissue protection.

The molecular data obtained at 200 mg/kg suggest that the therapeutic response was accompanied by changes in apoptosis-related protein expression. The 5-FU model group showed a pro-apoptotic pattern, with higher Bax and total caspase-3 and lower Bcl-2 abundance, whereas the BE group showed partial normalization of these markers. Because the molecular analyses were restricted to a single BE dose and were based on three biological samples per group, these results should be regarded as exploratory associations rather than evidence of a dose-dependent anti-apoptotic mechanism.

Inflammation-related markers showed a similar pattern. At the 200 mg/kg dose, *Tnf* and *Nos2* mRNA expression and iNOS protein abundance were lower in the BE group than in the 5-FU model group. These findings support an association between BE treatment and reduced inflammatory marker expression in colonic tissue. However, the current data do not define the upstream signaling pathways involved. Whether NF-κB or other transcriptional regulators mediate these changes requires targeted studies of pathway activation, barrier-related proteins, oxidative stress, and direct apoptotic indices.

The microbiota analysis provides an additional treatment-associated observation. The model group showed altered microbial diversity and community composition, whereas the BE 200 mg/kg samples displayed a community profile more similar to that of the control group. However, only three samples per group were analyzed, and LEfSe was used as an exploratory screening approach. These findings therefore do not establish that microbiota changes mediated the therapeutic response. Larger studies incorporating functional validation, microbial metabolite analysis, and causal approaches such as microbiota transfer will be needed to determine whether specific taxa contribute to injury or recovery.

The implications of the present findings may extend beyond 5-FU. The protective features identified here, particularly attenuation of inflammatory signaling and apoptosis, are pathophysiologically relevant to intestinal injury induced by other chemotherapeutic agents, including oxaliplatin and irinotecan [22,30,31]. Whether BE confers comparable protection against these agents remains unknown and warrants dedicated investigation. In addition, an intriguing possibility is that BE or related derivatives might not only reduce treatment-associated intestinal injury but also complement antitumor therapy. For example, betulinic acid has shown direct cytotoxic effects against colorectal cancer cells through mitochondrial apoptosis-related mechanisms [32,33]. This raises the possibility of a dual-action strategy in which intestinal protection and tumor sensitization could be integrated, although this concept will require rigorous validation in tumor-bearing models.

Our results are also broadly consistent with prior reports on other pentacyclic triterpenes. Oleanolic acid has been reported to ameliorate chemotherapy-associated intestinal injury through suppression of inflammatory signaling and restoration of antioxidant defense [34], whereas ursolic acid and glycyrrhizic acid have also shown tissue-protective activity in mucosal injury models [35,36]. These parallels suggest that BE may share a broader triterpene-associated protective profile. At the same time, BE has practical advantages, including abundance in birch bark, relative accessibility, and suitability as a scaffold for further derivatization [22,23,24].

Several limitations should be acknowledged. First, the group sizes were modest, particularly for the molecular and microbiota analyses, which were based on three biological samples per group. These analyses therefore had limited statistical power, and the reported p values, effect directions, and between-group patterns should be regarded as exploratory and hypothesis-generating rather than confirmatory. Larger, independently powered studies are required to validate the reproducibility and magnitude of these effects. Second, the molecular and microbiota analyses were restricted to the 200 mg/kg dose and therefore do not establish dose-dependent mechanisms. Third, although blinded semi-quantitative histopathological scoring and crypt-depth measurements were added, the scoring remains partly observer-dependent and the morphometric analysis depends on section orientation. Fourth, the microbiota findings are descriptive and do not establish causality. Fifth, the safety assessment was limited to short-term overt toxicity and did not include serum biochemistry or histological examination of major organs. Finally, no in vitro epithelial model was included to distinguish direct epithelial actions of BE from systemic effects observed in vivo. These limitations warrant cautious interpretation and validation in larger, independently powered studies.

Overall, BE treatment improved clinical, morphometric, and histopathological readouts in mice with 5-FU-induced intestinal mucositis. Exploratory analyses at 200 mg/kg also identified treatment-associated changes in apoptosis-related proteins, inflammatory markers, and gut microbial communities. These findings warrant further evaluation of BE in larger cohorts and in tumor-bearing models, with direct assessment of molecular targets, intestinal barrier function, microbiota–host interactions, and broader safety endpoints.

## 5. Conclusions

Within the limitations of this exploratory study, BE attenuated clinical and histopathological features of 5-FU-induced intestinal mucositis, and no overt adverse effects were detected in the short-term toxicity assessment. At 200 mg/kg, the therapeutic response was accompanied by lower *Tnf* and *Nos2* mRNA expression, lower iNOS, Bax, and total caspase-3 protein abundance, higher Bcl-2 protein abundance, and treatment-associated changes in gut microbial diversity and composition. These findings support further investigation of BE as a potential therapeutic candidate for 5-FU-associated intestinal mucositis but do not establish a definitive molecular or microbiota-mediated mechanism.

## Data Availability

All data supporting the findings of this study are available within the article and its Supplementary Materials. Further inquiries can be directed to the corresponding author(s).

## Supplementary Materials

The following supporting information can be downloaded at: https://www.mdpi.com/article/10.3390/nu18152487/s1, Figure S1. Representative gross images of major organs from mice in the short-term overt toxicity assessment; Figure S2. Quantification of colonic crypt depth; Figure S3. Western blot source data for Bax; Figure S4. Western blot source data for Bcl-2; Figure S5: Western blot source data for total caspase-3; Figure S6. Western blot source data for iNOS; Figure S7. Reference image of the prestained molecular-weight marker used for Western blot analysis; Table S1. Blinded scoring of H&E-stained section for safety assessment; Table S2. Blinded scoring of treated experimental H&E-stained section; Table S3. Primer sequences.

## Abbreviations

The following abbreviations are used in this manuscript:

CIM	Chemotherapy-induced intestinal mucositis
5-FU	5-Fluorouracil
BE	Betulin
DAI	Disease Activity Index
PCoA	Principal Coordinates Analysis
PCA	Principal Component Analysis
NMDS	Non-metric multidimensional scaling
LEfSe	Linear discriminant analysis effect size
H&E	Hematoxylin and eosin
*N**os**2*	Nitric oxide synthase 2
*T**nf*	Gene encoding tumor necrosis factor alpha
iNOS	Inducible nitric oxide synthase
