# Supplementary material for: Betulin Alleviates 5-Fluorouracil-Induced Intestinal Mucositis in Mice
Source: Nutrients. 2026 Aug 1;18(15):2487. doi: 10.3390/nu18152487 (PMC13468049; doi:10.3390/nu18152487)
Supplement: Supplementary file 1 [file nutrients-18-02487-s001.zip › nutrients-4392598-supplementary.pdf]

Supplementary materials

Betulin Alleviates 5-Fluorouracil-Induced Intestinal Mucositis in Mice

Shiyi Sun [1,†], Ziming Wang [1,†], Haoyue Xu [1], Chengheng Dai [1], Wenhannian Li [1], Mengcheng Ying [1], Ayimnisa Ayitniyaz [1], Changmin Shao [2,*] and Zhiwei Hu [1,*]

[1] National and Local Joint Engineering Research Center of Ecological Treatment Technology for Urban Water Pollution, Zhejiang Provincial Key Laboratory for Water Environment and Marine Biological Resources Protection, College of Life and Environmental Science, Wenzhou University, Wenzhou, 325035, China

[2] Wenzhou Institute, University of Chinese Academy of Sciences, Wenzhou, 325001, China

* Correspondence: changmin_shao@163.com (C.S.); zhiwei.hu@wzu.edu.cn (Z.H.)

† These authors contributed equally to this work.

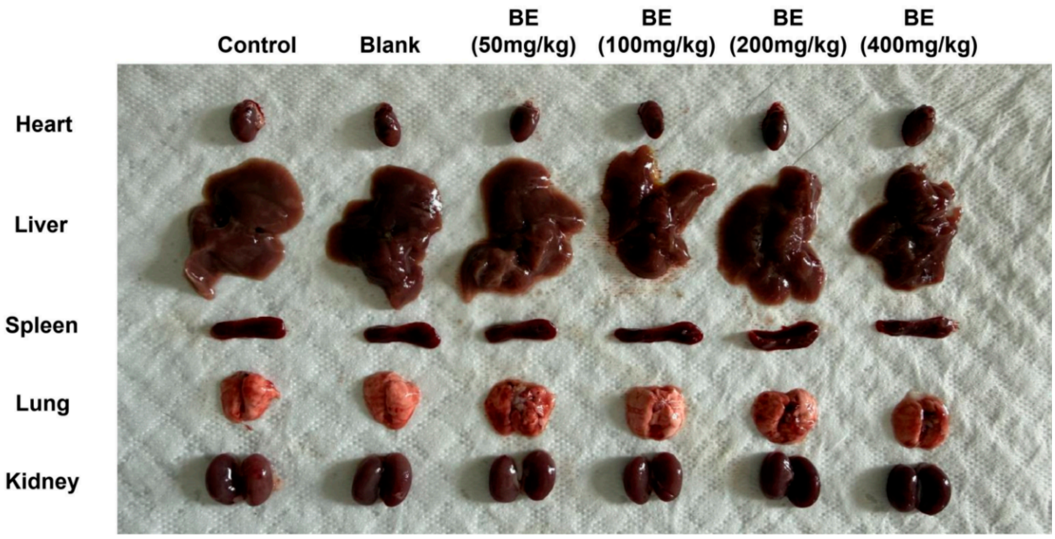

Figure S1. Representative gross images of major organs from mice in the short-term overt toxicity assessment.

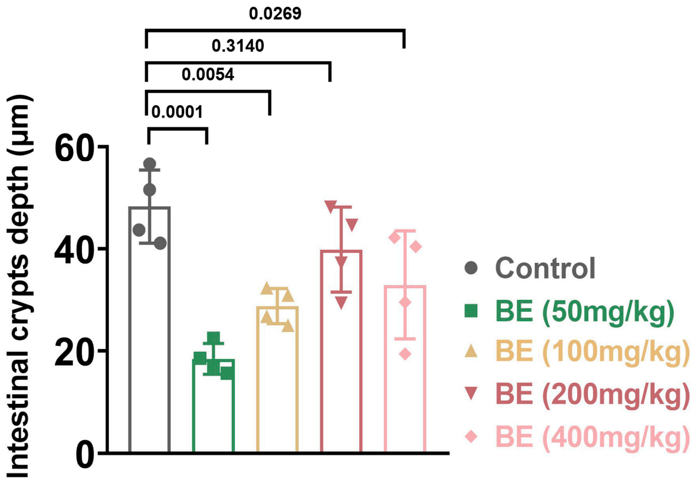

Figure S2. Quantification of colonic crypt depth. **Note:** Crypt depth could not be reliably quantified in the 5-FU model group because severe mucosal injury and crypt loss prevented the identification of intact, well-oriented crypts. The model group was therefore designated as not quantifiable and excluded from the crypt-depth statistical analysis.

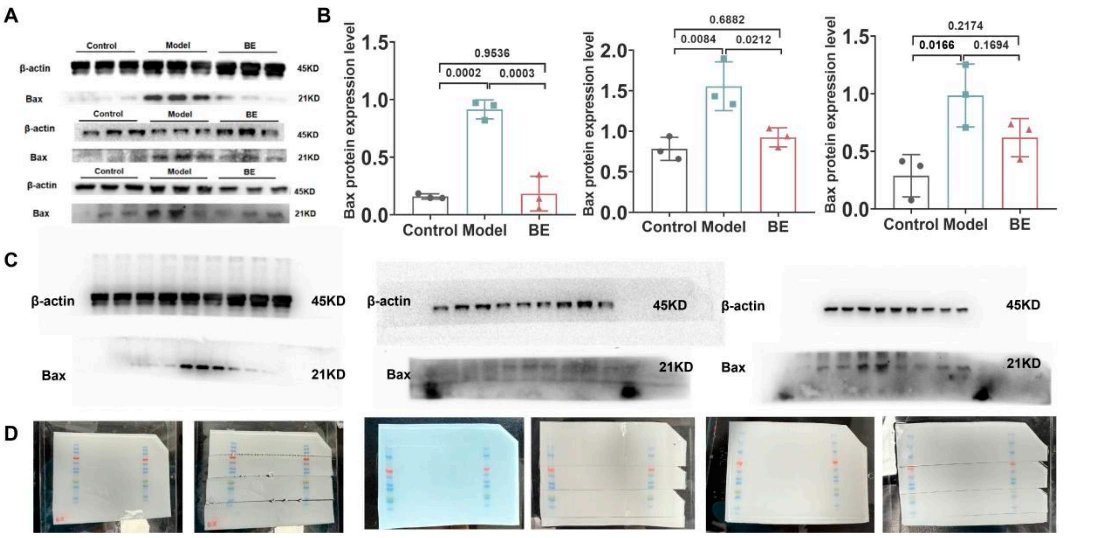

Figure S3. Western blot source data for Bax. (A) Representative Western blot bands for Bax and β-actin used in the main figure. (B) Relative Bax protein abundance obtained from each of the three technical runs. (C) Available original chemiluminescence imaging files. (D) Photographs of the corresponding membranes. Each technical run included three independent biological samples per group obtained from three individual mice.

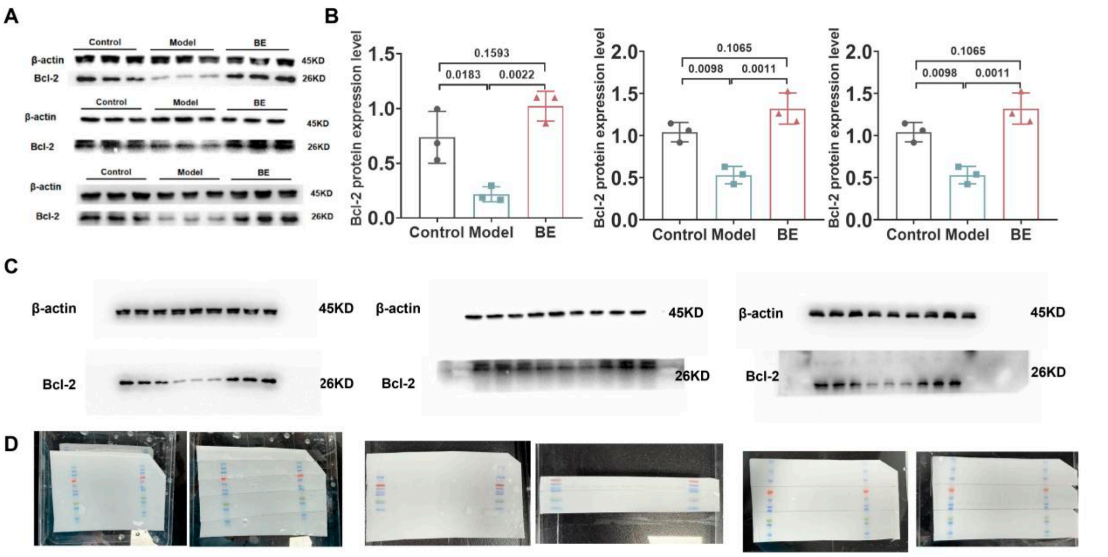

Figure S4. Western blot source data for Bcl-2. (A) Representative Western blot bands for Bcl-2 and β-actin used in the main figure. (B) Relative Bcl-2 protein abundance obtained from each of the three technical runs. (C) Available original chemiluminescence imaging files. (D) Photographs of the corresponding membranes. Each technical run included three independent biological samples per group obtained from three individual mice.

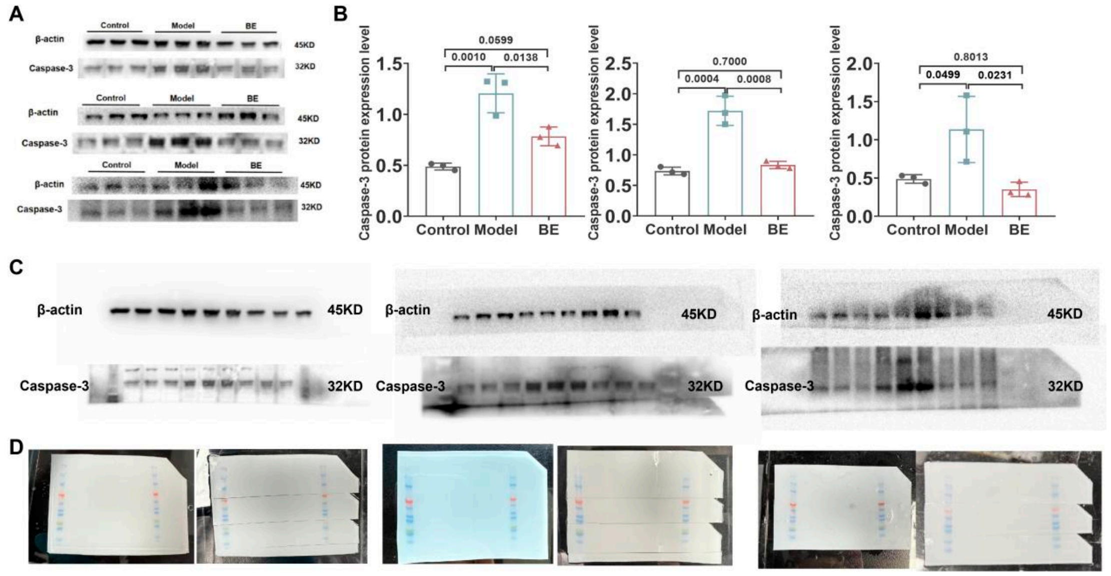

Figure S5. Western blot source data for total caspase-3. (A) Representative Western blot bands for total caspase-3 and β-actin used in the main figure. (B) Relative total caspase-3 protein abundance obtained from each of the three technical runs. (C) Available original chemiluminescence imaging files. (D) Photographs of the corresponding membranes. Each technical run included three independent biological samples per group obtained from three individual mice.

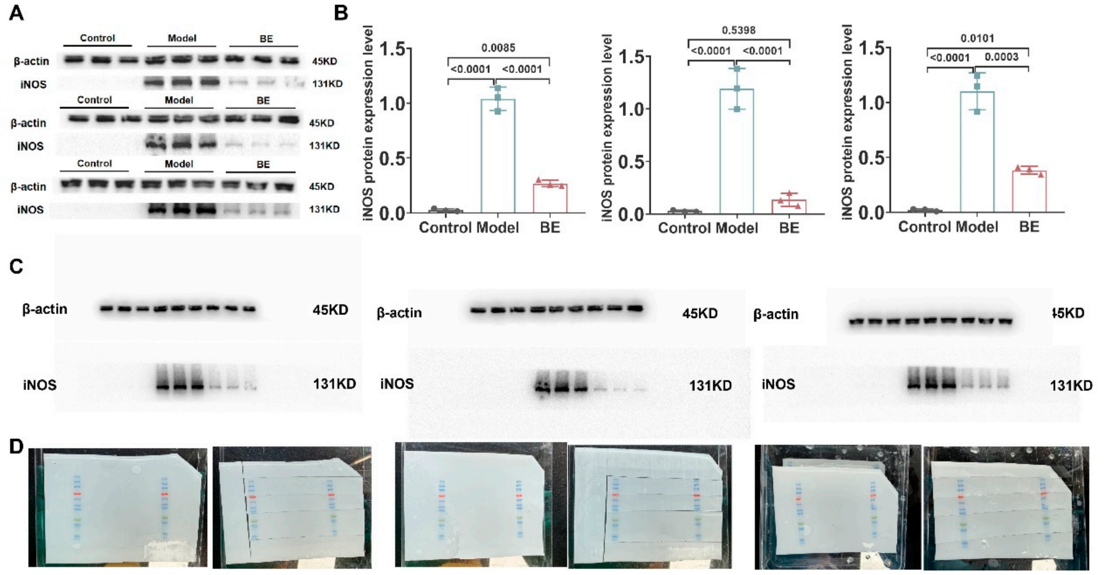

Figure S6. Western blot source data for iNOS. (A) Representative Western blot bands for iNOS and β-actin used in the main figure. (B) Relative iNOS protein abundance obtained from each of the three technical runs. (C) Available original chemiluminescence imaging files. (D) Photographs of the corresponding membranes. Each technical run included three independent biological samples per group obtained

from three individual mice.

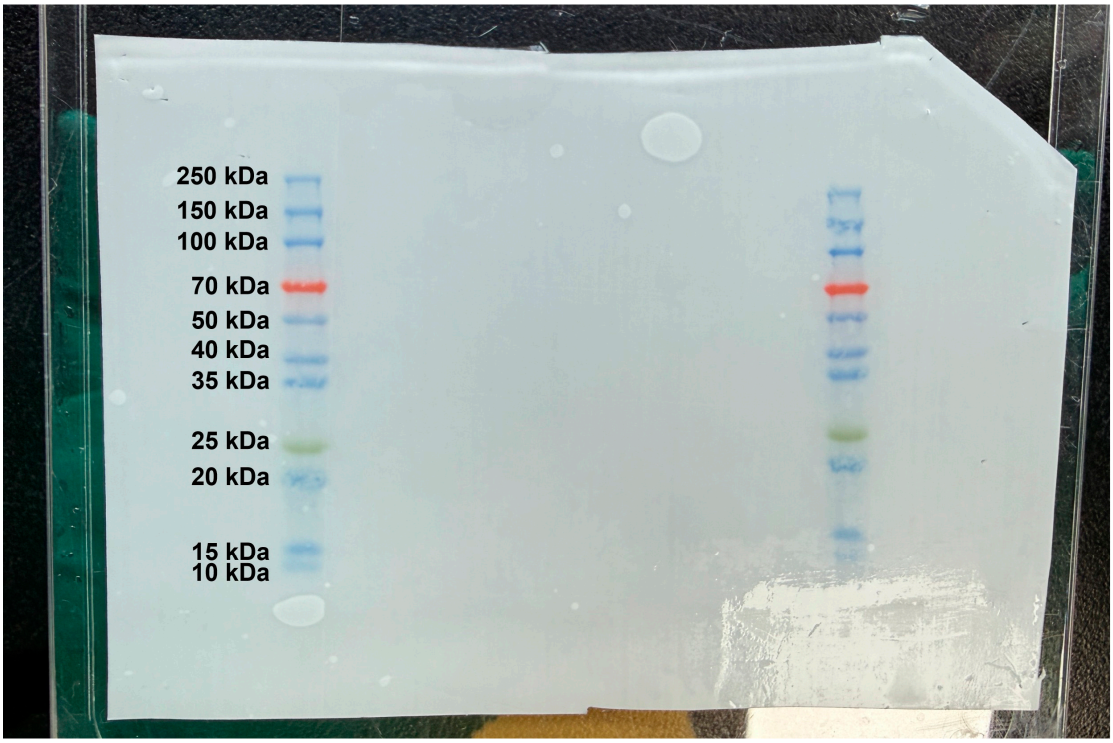

Figure S7. Reference image of the prestained molecular-weight marker used for Western blot analysis. Marker sizes are indicated in kilodaltons (kDa). This image is provided as a molecular-weight reference and was acquired separately from the chemiluminescence images shown in Fig. S3–S6.

Table S1. Blinded scoring of H&E-stained section for safety assessment.

	Index	Descriptive Score	Total points
Control	Crypt architectural distortion	1	4
	Crypt regeneration	0.5	
	Crypt loss	0.5	
	Luminal sloughing of cellular debris	0	
	Lamina propria inflammatory cell infiltrate	2	
	Mucosal ulceration	0	
Blank	Crypt architectural distortion	0.5	4.5
	Crypt regeneration	0.5	
	Crypt loss	1	
	Luminal sloughing of cellular debris	0.5	
	Lamina propria inflammatory cell infiltrate	2	
	Mucosal ulceration	0	
BE (50mg/kg)	Crypt architectural distortion	0.5	5
	Crypt regeneration	1	
	Crypt loss	0.5	
	Luminal sloughing of cellular debris	0	
	Lamina propria inflammatory cell infiltrate	3	
	Mucosal ulceration	0	
BE (100mg/kg)	Crypt architectural distortion	0	5.5
	Crypt regeneration	1	
	Crypt loss	0.5	
	Luminal sloughing of cellular debris	0	
	Lamina propria inflammatory cell infiltrate	4	
	Mucosal ulceration	0	
BE (200mg/kg)	Crypt architectural distortion	1	4
	Crypt regeneration	0	
	Crypt loss	0	
	Luminal sloughing of cellular debris	0	
	Lamina propria inflammatory cell infiltrate	3	
	Mucosal ulceration	0	
BE (400mg/kg)	Crypt architectural distortion	1	5
	Crypt regeneration	0.5	
	Crypt loss	1	
	Luminal sloughing of cellular debris	0.5	
	Lamina propria inflammatory cell infiltrate	2	
	Mucosal ulceration	0	

Table S2. Blinded scoring of treated experimental H&E-stained section.

	Index	Descriptive Score	Total points
Control	Crypt architectural distortion	0.5	3.5
	Crypt regeneration	0	
	Crypt loss	0.5	
	Luminal sloughing of cellular debris	0.5	
	Lamina propria inflammatory cell infiltrate	2	
	Mucosal ulceration	0	
Model	Crypt architectural distortion	4	19
	Crypt regeneration	2	
	Crypt loss	3	
	Luminal sloughing of cellular debris	3	
	Lamina propria inflammatory cell infiltrate	3	
	Mucosal ulceration	4	
BE (50mg/kg)	Crypt architectural distortion	0.5	6
	Crypt regeneration	0.5	
	Crypt loss	1	
	Luminal sloughing of cellular debris	0.5	
	Lamina propria inflammatory cell infiltrate	3	
	Mucosal ulceration	0.5	
BE (100mg/kg)	Crypt architectural distortion	0.5	6.5
	Crypt regeneration	2	
	Crypt loss	1	
	Luminal sloughing of cellular debris	1	
	Lamina propria inflammatory cell infiltrate	2	
	Mucosal ulceration	0	
BE (200mg/kg)	Crypt architectural distortion	0.5	4
	Crypt regeneration	1	
	Crypt loss	0.5	
	Luminal sloughing of cellular debris	1	
	Lamina propria inflammatory cell infiltrate	1	
	Mucosal ulceration	0	
BE (400mg/kg)	Crypt architectural distortion	0.5	5.5
	Crypt regeneration	2	
	Crypt loss	0	
	Luminal sloughing of cellular debris	1	
	Lamina propria inflammatory cell infiltrate	2	
	Mucosal ulceration	0	

Table S3. Primer sequences.

Gene	Forward primer(5'-3')	Reverse primer(5'-3')
Tnf	CCCTCACACTCAGATCATCTTCT	GCTACGACGTGGGCTACAG
Nos2	GTTCTCAGCCCAACAATACAAGA	GTGGACGGGTCGATGTCAC
Gapdh	AGGTCGGTGTGAACGGATTTG	TGTAGACCATGTAGTTGAGGTCA
